# Alpha toxin production potential and antibiotic resistance patterns of *clostridium perfringens* isolates from meat samples

**DOI:** 10.5713/ab.24.0210

**Published:** 2024-06-25

**Authors:** Tehreem Ali, Arslan Sarwar, Aftab Ahmad Anjum

**Affiliations:** 1 Department of Microbiology, University of Central Punjab, Lahore 54000, Pakistan; 2 Institute of Microbiology, University of Veterinary and Animal Sciences, Lahore 54000, Pakistan

**Keywords:** *Clostridium perfringens*, Foodborne Illness, Molecular Characteristics, Toxinotyping and Antibiotic Resistance

## Abstract

**Objective:**

This research aimed to analyze the prevalence, molecular characteristics, toxinotyping, alpha toxin production potential, and antibiotic resistance pattern of *Clostridium perfringens* (*C. perfringens*) isolates in meat samples collected from various sources.

**Methods:**

Sixty meat samples were screened for alpha toxin using enzyme-linked immunosorbent assay, revealing a positivity rate of 13.3%, predominantly in raw poultry meat. Subsequent culturing on Perfringens agar identified nine samples harboring characteristic *C. perfringens* colonies, primarily isolated from raw poultry meat. Molecular confirmation through 16S rRNA gene amplification and sequencing authenticated twelve isolates as *C. perfringens*, with nine strains exhibiting genetic resemblance to locally isolated strains. Toxinotyping assays targeting alpha toxin-specific genes confirmed all nine isolates as type A *C. perfringens*, with no detection of beta or epsilon toxin genes. Hemolytic assays demonstrated varying alpha toxin production potentials among isolates, with accession number OQ721004.1 displaying the highest production capacity. Moreover, antibiotic resistance profiling revealed multi-drug resistance patterns among the isolates.

**Results:**

The study identified distinct clusters within *C. perfringens* strains, indicating variations. Phylogenetic analysis delineated genetic relatedness among strains, elucidating potential evolutionary paths and divergences.

**Conclusion:**

The findings underscore the need for robust surveillance and control measures to mitigate the risk of *C. perfringens* contamination in meat products, particularly in raw poultry meat. Enhanced monitoring and prudent antimicrobial stewardship practices are warranted in both veterinary and clinical settings to address the observed antibiotic resistance profiles and prevent foodborne outbreaks.

## INTRODUCTION

*Clostridium perfringens* (*C. perfringens*) is a significant cause of human gastrointestinal disorders including food poisoning, diarrhea brought on by antibiotics and nosocomial diarrheal illness [1,2]. These bacteria are ubiquitous in nature and are more widely present than any other bacteria. *C. perferingens* is reported to be present in a variety of foods but the meat industry is primarily concerned about it because of its predilection for the amino acids in meat. In Germany, UK and Canada, *C. perferingens* has been reported to be the major cause of food poisoning [3,4].

The bacteria may grow and remain in food due to their capacity to create spores and their quick growth levels at a variety of temperatures. Moreover, *C. perferingens* does not have the ability to produce 13 of its amino acids and protein rich food is a source of all these amino acids for its growth. Most *C. perfringens* food poisoning incidents have happened at institutions and food service businesses that prepare a lot of food ahead of time for serving [5].

The number of bacteria in a meal grows quickly if it is either overcooked or undercooked. Most people do not call health authorities because of the very mild nature of the illness and the relatively brief duration of the symptoms [6]. Although *C. perfringens* food poisoning is not a disease that needs to be reported, and the number of cases is likely significantly understated, enough outbreaks are recorded to make it one of the most prevalent foodborne illnesses in developed countries [7].

At least 17 distinct soluble antigens, often known as toxins, are produced by *C. perfringens* and may have a role in pathogenesis. It’s unknown if any of these antigens directly causes lesions or contributes to the pathogenesis of humans or animals. Nonetheless, these antigens are frequently referred to as “toxins” [8]. These toxins are produced by strains of *C. perfringens* of all five kinds, but type A strains often produce the most of it. Among the perfringens toxins, alpha toxin has been investigated the most and has been shown to possess phospholipase C as well as fatal, necrotizing, hemolytic, and cytolytic properties [9].

A large number of diseases are assumed to be caused by these toxins of *clostridium perferingens* [10]. Important toxins of *C. perferingens* include alpha, beta, epsilon, and iota toxins. Total 2% to 5% of *C. perferingens* produce cpe toxin, most of which are *clostridium perferingens* type A. Different routes could be involved in the contamination of meat with clostridium perferingens. This paper explains the *clostridium perferingens* outbreak in meat, toxinotyping, molecular characterization, alpha toxin production potential and antibiotic resistance of *C. perferingens* [11]. This research on *C. perferingens* in meat samples addresses critical gaps in understanding foodborne pathogen prevalence and characteristics, particularly in raw poultry meat. By analyzing molecular features, toxin production potential, and antibiotic resistance patterns, the study offers insights crucial for enhancing food safety measures and mitigating the risk of contamination-related outbreaks. Its findings underscore the necessity for vigilant surveillance and informed antimicrobial stewardship to safeguard public health.

## MATERIALS AND METHODS

The study was conducted at the University of Veterinary and Animal Sciences Lahore. The poultry meat samples were divided into three categories as raw poultry meat samples, processed poultry meat samples and poultry meat products.

### Toxin analysis by enzyme-linked immunosorbent assay

Similar to Clostridium was cultured from meat sample and sample was prepared containing pure toxin [12]. Antibody of interest was coated in enzyme-linked immunosorbent assay (ELISA) plate. The sample was incubated and then washed to remove unbound proteins. After that secondary antibody was added followed by addition of substrate specific to that antibody and then the wavelength was measured by spectrophotometer and amount of toxin present was calculated.

### Laboratory identification of *C. perfringens*

Pre-enrichment of sample was done in peptone broth and after that selective enrichment was done in fluid thioglyocolate Broth. It was then cultured on RCM agar. Confirmation was done by Gram staining, biochemical tests and lecithinase test [13].

### Molecular confirmation of *C. perfringens* identification

The molecular confirmation of the *C. perfringens* was done for identification and confirmation of bacterium. DNA was isolated or extracted from the suspected sample by using commercially available extraction kits. As the DNA is extracted polymerase chain reaction (PCR) technique used to amplify the specific *C. perfringens* genome [14]. Specific primers were designed to identify *C. perfringens* gene sequences. Amplification of specific targeted DNA was done by performing thermal cycling through multiple steps (denaturation, annealing and extension). PCR products were analyzed using gel electrophoresis in which the DNA fragments were separated according to molecular size and then the specific amplicon was visualized under UV light for further confirmation [15].

### Toxinotyping of *C. pefringens*

Toxinotyping was performed for the identification of *C. perfringens* on the basis of toxins they produce [3]. The toxinotype was determined by using specific primers for the alpha, beta and epsilon toxin produced by *C. perfringens*.

### Phylogenetic analysis

To sequence the DNA amplicons, Sanger dideoxy sequencing was employed. For retrieval, the FASTA format was used. Following cleaning with the JUST bio program, the sequences were analyzed with the Basic Local Alignment Search Tool (BLAST-n) to find regions of high similarity. Additionally, accession numbers were obtained for these sequences, and they were submitted to GenBank NCBI. Using the MEGA-X program, nine *C. perfringens* isolates’ sequencing data were combined with *C. perfringens* sequences isolated from Pakistan and worldwide from GenBank NCBI to generate a phylogenetic tree.

### Alpha toxin production potential of *C. perfringens* type A

By the hemolytic assay, the alpha toxin production potential for each identified type A C. perfringens toxin was determined after 24, 48, and 72 hours of broth culture. After 24 hours 100 μL of broth culture was mixed with sheep red blood cells in 96 well plate and incubated for 30 to 40 mins at 37°C and values were recorded at 630 nm. The same procedure was repeated after 48 and 72 h.

### Antibiotic resistance pattern of *C. perfringens*

Antibiotic resistance pattern was determined by performing Kirby-Bauer method (Disc diffusion method). In accordance with the Clinical and Laboratory Standards Institute (CLSI) 2020 guide, culture sensitivity testing was carried out using the Kirby-Bauer method for antibiotics such as ampicillin, cloxacillin, amoxicillin, ceftriaxone, cefoxitin, levofloxacin, tetracyclin, doxycycline, oxytetracyclin, kanamycin, gentamycin, neomycin, streptomycin, bacitracin, chloramphenicol, norfloxacin and ciprofloxacin. The optical density was adjusted to 0.1 at 630 nm to inoculate the *C. perfringens* cultures into 0.5 McFarland. Antibiotic discs were administered using a disc dispenser after the prepared inoculum cultures had spread out on nutrient agar plates. Following a 24-hour incubation period at 37°C, a ruler was used to measure the clear zone of inhibition’s size in millimeters. By comparing the zones of inhibition to the CLSI-2020 manual, they were categorized as sensitive, resistant, or intermediate [16].

### Statistical analysis

Data was analyzed through one way analysis of variance followed by Duncan’s multiple range test as post hoc by using Statistical package for social sciences (SPSS) Version 20.0 and level of significance (p-value) of 0.05 was chosen for statistical analysis.

## RESULTS

### Toxin analysis by enzyme-linked immunosorbent assay

Firstly, the collected meat samples were screened by ELISA to detect alpha (α) toxin. The ELISA results showed that out of sixty tested samples, only eight (8) were positive for α toxin. Out of 8 positive samples, 7 samples were from raw poultry meat and one positive sample was from the meat by product while no sample was positive for α toxin from processed meat.

### Identification of *C. pefringens*

All the collected samples were primarily cultured on Perfringens agar (Himedia laboratories, Thane, India). Black, slightly raised smooth bacterial colonies under anaerobic conditions were observed in nine (9) samples. Microscopically gram-positive rods with curved ends and oval shaped sub-terminal endospores were observed by gram staining and spore staining, respectively. From isolated bacteria, 12 out of 25 isolates were considered *C. pefringens* based upon culture, microscopic and biochemical characteristics among these highest number of isolates (9) were from raw poultry meat samples followed by poultry meat by products (2) and lowest from processed poultry meat samples (1).

### Molecular confirmation of *C. pefringens* isolates

**Figure 1:**
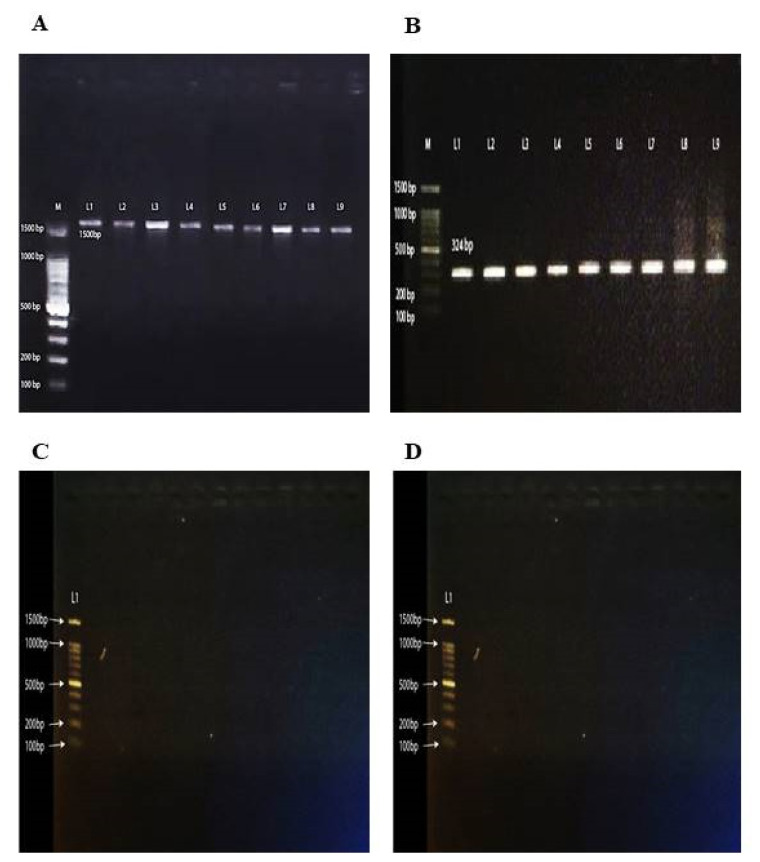
The PCR confirmation of *Clostridium perferingens* isolates and toxin genes detection using PCR. This figure demonstrates the results of PCR analyses used to confirm the presence of *C. perfringens* isolates and to detect specific toxin genes. (A) Shows successful amplification of the 16S rRNA gene in lanes 1–9, confirming the presence of *C. perfringens*. (B) Displays the amplification of the alpha toxin gene in lanes 1–9. (C and D) Show that there was no amplification for the epsilon and beta toxin genes, respectively, indicating these genes were not present in the tested isolates. M: DNA ladder,100 bp. PCR, polymerase chain reaction.

Afterwards, biochemically characterized *C. perferingens* isolates (n = 12) were confirmed by targeting 16S rRNA gene amplification by PCR followed by nucleotide sequence analysis. The purity of the extracted DNA from fresh broth cultures of *C. perfringens* isolates was assessed by Nanodrop (ThermoFisher Scientific, London, UK) and the highest concentration of extracted DNA was 440 ng/μL and lowest 9.8 ng/μL. Amplification of 16S rRNA gene revealed 1,500 base pair band on gel electrophoresis visualized by gel doc at 260 nm wavelength (Figure 1A). Original FASTA files of amplified DNA products sequenced by Sanger dideoxy method were reviewed for sequence alignment using n-Blast ( https://blast.ncbi.nlm.nih.gov/Blast.cgi?PROGRAM=blastn&BLAST_SPEC=GeoBlast&PAGE_TYPE=BlastSearch ) following cleaning, by Just tool (JustBio, Ottawa, ON, Canada). After confirmation all sequences were submitted to NCBI for accession numbers and based upon nucleotide sequence sequences nine (9) were of *C. perfringens*. The accession Numbers allocated by GenBank for characterized *C. perfringens* isolates were OQ720996.1, OQ720997.1, OQ7209978.1, OQ720999.1, OQ721000, OQ721001.1, OQ721002.1, OQ721003.1, and OQ721004.1.

### Toxinotyping of *C. pefringens*

Confirmation of the *C. perfringens* isolates for toxin typing was carried out by targeting α toxin specific gene by PCR. All of the nine isolates which were confirmed as *C. perfringens* by 16S rRNA nucleotide sequencing were positive for α toxin gene and a band of 324 bps was visualized by Gel Doc (Figure 1B). None of the isolates showed beta and epsilon toxin gene (Figure 1C, 1D). Based upon toxin gene specific amplification all of the nine isolates were recorded as *C. perfringens* type A as other toxin genes were not amplified by PCR.

### Phylogenetic analysis

**Figure 2:**
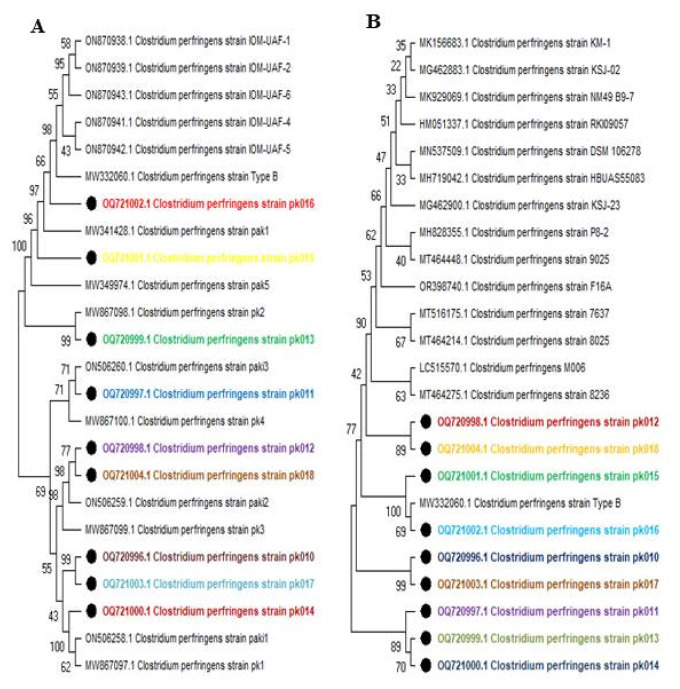
Phylogenetic analysis of genomic sequences. This figure presents the phylogenetic analysis of the 16S rRNA gene sequences from *Clostridium perfringens* strains. (A) depicts the phylogenetic relationships among locally isolated *C. perfringens* strains, indicating their genetic similarity and diversity within the local context. (B) compares these local strains with internationally isolated strains, showing the broader evolutionary relationships and genetic diversity on a global scale. The phylogenetic trees were constructed using sequence alignment and appropriate tree-building algorithms, providing a visual representation of the genetic distances and evolutionary history of the analyzed strains (local strains indicated with bold circles).

Upon conducting a phylogenetic analysis of the 16S ribosomal RNA gene sequences of *C. pefringens*, the phylogenetic analysis of locally isolated strains revealed distinct groupings depending upon genetic diversion (Figure 2A). An in-group comprising most strains and an out-group including local strains ON506260.1, MW867100.1, ON506259.1, MW 867099.1, ON506258.1, and subject strains OQ720997.1, OQ720998.1, OQ721004.1, and OQ720996.1. Within the group, subject strain pk016 exhibited 98% genetic resemblance to locally isolated type B strains within the clade. Subject strain OQ721002.1 showed 99% genetic similarity to local strain MW867098.1. Subject strains OQ720996.1 displayed 99% similarity, while OQ721000.1 demonstrated 100% genetic relatedness to local strains ON506258.1 and MW867097.1.

Bootstrap analysis, used to assess the robustness of the tree’s topology, indicated varying degrees of genetic resemblance among the strains. Within the in-group, subject strains OQ720998.1 and OQ721004.1 exhibited an 89% genetic similarity. Similarly, subject strains OQ720998.1 and OQ 721003.1 showed a genetic resemblance of up to 99%. Overall, the phylogenetic analysis suggested a strong genetic resemblance among the subject strains, and also some degree of relatedness to internationally reported strains (Figure 2B).

**Figure 3:**
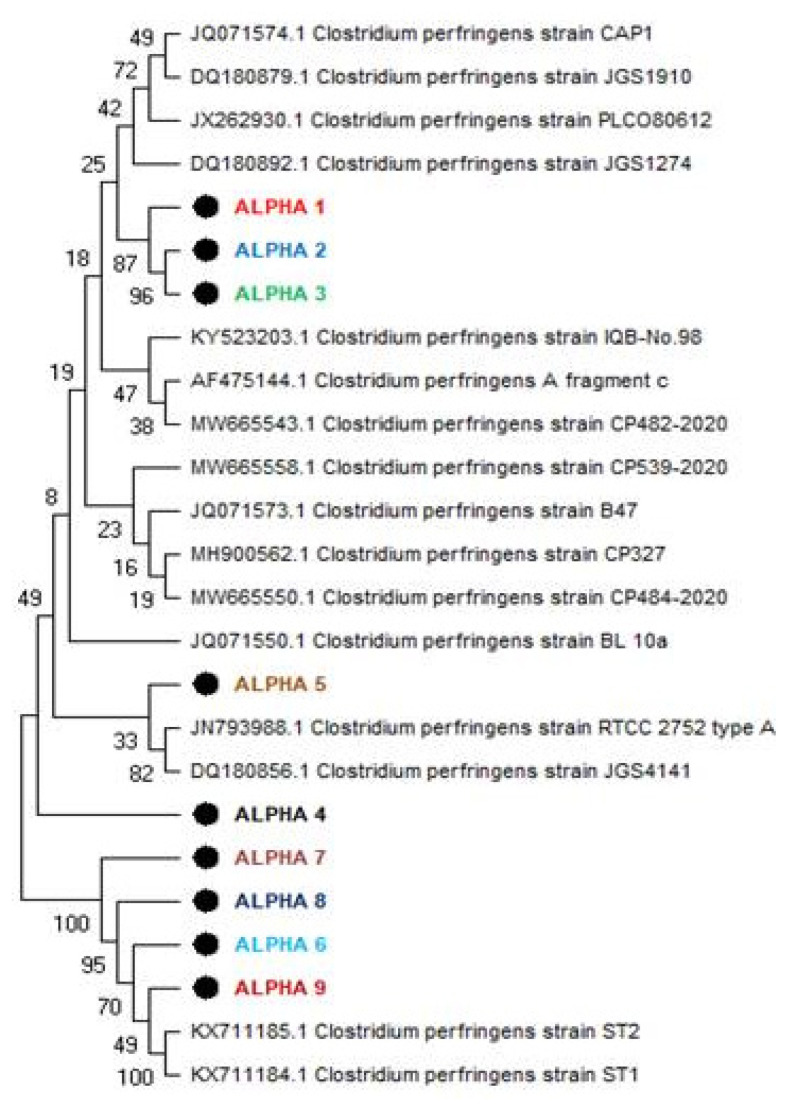
Phylogenetic analysis of alpha toxin producing genes of *Clostridium perfringens* strains. This figure illustrates the phylogenetic analysis of alpha toxin-producing genes from *C. perfringens* strains. This figure depicts the phylogenetic relationships among locally isolated strains, with each strain represented by a bold circle, highlighting their genetic similarity and diversity within the local context.

The phylogenetic analysis of alpha toxin-producing genes in *C. pefringens* strains provides valuable insights into the genetic relatedness and evolutionary history of these strains (Figure 3). The identification of an in-group comprising most strains and an out-group including specific research strains, ALPHA7, ALPHA8, ALPHA9, and ALPHA6, alongside previously reported strains KX711185.1 and KX711184.1 isolated in China in 2016, suggests a potential divergence in evolutionary paths among these clusters. Within the in-group, the observed genetic resemblances among subject strains ALPHA1, ALPHA2, and ALPHA3, underscore their close evolutionary ties, indicative of a shared genetic heritage or recent common ancestor as ALPHA1 exhibited an 87% genetic resemblance to strains ALPHA2 and ALPHA3, while ALPHA2 and ALPHA3 showed a 96% genetic similarity. Conversely, the lower genetic similarity of about 33% was observed for ALPHA5 compared to reported strains JN793988.1 type A isolated in Iran and DQ180856.1 isolated in North America in 2006 suggests a more distant evolutionary relationship, potentially indicative of genetic divergence or independent evolutionary trajectories.

### Alpha toxin production potential

Alpha toxin production potential for each of the confirmed *C. perfringens* toxin type A was calculated at 24, 48, and 72 hours of broth culture by hemolytic assay (Table 1). At 24 hours of incubation isolate with accession number OQ721004.1 produced the highest mean alpha hemolytic units (7.33±0.47) and isolate OQ720997.1 the lowest (4.33±0.47). Statistically, mean alpha hemolytic units of isolate 7.33±0.47 differed significantly with means of other tested isolates post 24 hours of incubation. At 48 hours of incubation isolate with accession number OQ721000.1 and OQ721004.1 produced equal and highest mean alpha hemolytic units (9.66±0.47) and isolate OQ7209978.1 the lowest (7.33±0.47). Statistically, mean alpha hemolytic units of isolates OQ721000.1 and OQ721004.1 differed non-significantly whereas significantly with rest of the tested isolates for alpha toxin production. Alpha toxin production potential for *C. perfringens* toxin type A at 24, 72 hours of broth culture by Hemolytic assay was the highest (10.66±0.47) by Accession number OQ721004.1 and the lowest (7.66±0.47) by the isolate OQ7209978.1. Statistically, mean alpha hemolytic units of isolate OQ721004.1 differed significantly with means of other tested isolates post 72 hours of incubation. Overall isolate OQ721004.1 exhibited the highest alpha toxin production potential among all of the tested isolates.

### Antibiotic resistance pattern of isolated *C. perfringens*

Antibiotic resistance pattern of characterized C. perfringens Type A isolates (n = 9) showed mean Test zone of inhibition of Ampicillin ranged between 14±1.73 and 5.66±0.57 being highest against isolate OQ721000.1 and the lowest in case of isolate OQ720997.1 (Table 2). By comparing with standard zone of inhibition of Ampicillin (≥17) all the nine isolates tested were declared resistant to ampicillin. The highest zone of inhibition (15.33±0.57) of Cloxacillin was recorded against Isolate OQ721001.1 and the lowest (8.66±0.57) for isolate OQ721000.1. In comparison with standard zone of inhibition of Cloxacillin (≥17) all of the tested isolates were resistant to Cloxacillin. In the case of Amoxicillin, the highest test zone of inhibition (18.66±0.57) recorded was against isolate OQ7209978.1 and the lowest (9.33±0.57) for isolate OQ721002.1. According to the standard zone of inhibition of amoxicillin (≥18), out of nine tested isolates two isolates (OQ7209978.1 and OQ720999.1) were recorded as sensitive and rest seven as resistant. Based upon interpretation of test zone of inhibitions with standard zone of inhibitions, isolate OQ720996.1 was sensitive to four antibiotics (Ceftriaxone, Bacitracin, Chloramphenicol, and Norfloxacin) and resistant to other tested antibiotics. Out of eighteen tested antibiotics Isolate OQ720997.1 was sensitive to only Bacitracin and Ciprofloxacin. Isolate OQ7209978.1 was sensitive to Ceftriaxone, Amoxycillin, and Ciprofloxacin, Isolate OQ720999.1 to Amoxycillin and Levofloxacillin, isolates OQ721000.1 and OQ721003.1 to Norfloxacin and Ciprofloxacin whereas isolates OQ721001.1, OQ721002.1 and OQ721004.1 to only Ciprofloxacin. Only two isolates were resistant against Ciprofloxacin the rest of the seven isolates were sensitive. Based upon the antibiotic resistance pattern out of nine tested isolates eight were multi-drug resistant.

## DISCUSSION

Although CPE-producing strains *C. perfringens* type A have been associated for at least 15 years to both food-borne and non-food-borne gastrointestinal disorders in humans, the pathophysiology of these illnesses is still not fully known. Our goal in this study was to isolate *C. perfringens* strains from meat samples and identify by microscopic, biochemical and molecular methods. A study conducted by Haider et al [17] isolated 34 *C. perfringens* type A strains from 134 intestinal samples which reveled 25.34% prevalence. Bangladesh has a 34.5% overall prevalence of *C. perfringens* in meat, while in several other nations especially Jordan and Egypt it is more than 40% [10].

Current study revealed 9 samples positive for *C. perfringens* type A confirmed by 16S rRNA and specie specific PCR while the total no of bacteria isolated from meat samples were 25. A study showed that 26 isolates (15.1%) were identified as *Clostridium* spp. based on phenotypic analysis. Using a species-specific 16S rRNA gene, PCR was used to identify 10 isolates as *C. perfringens*. Multiplex PCR results showed that the cpa gene resulting *C. perfringens* toxin type A was most prevalent (90%) among the isolates, followed by the cpe gene (caused by type F). No isolates carried the cpb, etx, or iap genes [18]. They observed prevalence rate of 25.37% of *C. perfringens* strains that were isolated from 134 samples. Since they tested positive for alpha toxin (CPA) and negative for other toxins including beta (CPB), epsilon (ETX), iota (ITX), enterotoxin (CPE), toxin perfringens large (TpeL), and necrotic B-like toxin (NetB), all of the isolated strains had toxinotype A [19].

To find out if any minor virulence genes were present, 84 isolates that were taken from flocks of sheep and goats were analyzed and sequenced. The findings revealed that 79 out of 84 isolates (94%), 29 out of 84 isolates (35%) had cpe detected, 28 out of 84 isolates (33%) had confirmed TpeL presence, and none of the isolates had the netB gene [20].

After comparing the subject strains with locally isolated strains using a phylogenetic analysis of the 16S ribosomal RNA gene sequences of *C. perfringens*, the resulting tree showed strains represented by accession numbers at terminal nodes, with entities or taxa connected through divergent points or internal nodes. The genetic separation between strains was represented by branch lengths. The complete *C. perfringens* population in the current study was divided into two main clusters by the neighbor joining tree, which was built using bootstrap values computed from 1,000 replications. The average pairwise distance between these clusters was 0.016 to 0.019. All of the cpe+ve isolates were in cluster I, while all of the cpe-ve isolates were in cluster II [21].

In past studies, there was a controversy in the conservancies of alpha toxin. Regardless of the species from which *C. perfringens* originated, the segregation pattern showed that the sequences randomly clustered into different clusters. This is consistent with the previous findings that the cpa gene of *C. perfringens* is highly conserved and that diversification is only observed at the interspecies level. The present sequence, however, clustered with the broiler isolate of *C. perfringens* (GU581186) from Iran. Thus, it was determined that the toxin type and the cpa gene genotype disagreed, and that the toxin typing of the *C. perfringens* strains had nothing to do with each other’s evolutionary history [22].

In current study, all the isolates were declared as resistant to ampiciline and cloxacilline while all the isolates were somehow sensitive to amoxicillin. Some isolates were sensitive to ceftriaxone, bacitracin, chloramphenicol and norfloxacin. Only one isolate showed sensitivity against ciprofloxacin, bacitracin and levofloxacin. Results revealed that Tetracycline resistance gene (41.33%) was found to be more prevalent than erythromycin (34.66%) and bacitracin (17.33%), according to antimicrobial resistance gene profiling [23]. In 2020 a study conducted in Punjab, Pakistan showed that all isolates (100%) were sensitive to rifampin and ceftiofur; the majority (57%) was sensitive to teicoplanin, chloramphenicol, amoxicillin, linezolid and enrofloxacin. A lower proportion of isolates (43%) were sensitive to ciprofloxacin and only 14% were susceptible to erythromycin [24]. One study found antimicrobial resistance profile of *C. perfringens* isolates obtained from various sources showed that, according to phenotypic and genotypic detection methods, the strongest resistance was observed against tetracycline (41.3%) and sulphonamides (76.8%) [25].

## CONCLUSION

This research provides comprehensive insights into the prevalence, molecular characteristics, toxinotyping, and antibiotic resistance patterns of *C. perfringens* in meat products. The findings underscore the urgent need for robust surveillance and control measures to mitigate the risk of contamination, particularly in raw poultry meat, to prevent foodborne outbreaks. Additionally, prudent antimicrobial stewardship practices are imperative in both veterinary and clinical settings to address the observed multi-drug resistance patterns and ensure effective therapeutic interventions.

## Figures and Tables

**Table 1:** Alpha toxin production potential of *Clostridium perfringens* type A at different incubation times by hemolytic assay

Accession No.	24 h culture	48 h culture	72 h culture
OQ720996.1	5.67±0.47^cd^	8.66±0.47^bc^	8.66±0.47^ab^
OQ720997.1	4.33±0.47^a^	8.66±0.47^bc^	8.66±0.47^ab^
OQ7209978.1	5.33±0.47^bc^	7.33±0.47^a^	7.66±0.47^a^
OQ720999.1	6.66±0.47^de^	8.33±0.47^ab^	8.66±0.47^ab^
OQ721000.1	6.66±0.47^de^	9.66±0.47^c^	0.33±0.47^cd^
OQ721001.1	6.33±0.47^cde^	8.66±0.47^bc^	9.33±0.47^bc^
OQ721002.1	4.66±0.47^ab^	7.33±0.47^a^	8.66±0.47^ab^
OQ721003.1	6.0±0.0^cd^	8.33±0.47^ab^	9.33±0.47^bc^
OQ721004.1	7.33±0.47^e^	9.66±0.47^c^	10.66±0.47^d^

a–e Means within a column having different letters differ (p<0.05).

**Table 2:** Mean zone of inhibitions of selected antibiotics against *Clostridium perfringens* type A isolates

Antibiotics	Mean zone of inhibition [20] of different isolates								

	OQ720996.1	OQ720997.1	OQ7209978.1	OQ720999.1	OQ721000.1	OQ721001.1	OQ721002.1	OQ721003.1	OQ721004.1
Ampicillin	8.33±0.57^b^	5.66±0.57^a^	9.66±0.57^bc^	10.66±0.57^c^	14±1.73^e^	12.66±0.57^de^	13.33±0.57^de^	10.66±0.57^c^	12.33±0.57^d^
Cloxacillin	9.66±0.57^b^	14.33±0.57^e^	12.66±0.57^cd^	11.66±0.57^c^	8.66±0.57^a^	15.33±0.57^f^	13.33±0.57^de^	11.66±0.57^c^	13.66±0.57^de^
Amoxicillin	13.66±0.57^d^	15.66±0.57^e^	18.66±0.57^g^	17.66±0.57^f^	11.66±0.57^c^	12.66±0.57^c^	9.33±0.57^a^	10.33±0.57^b^	11.66±0.57^c^
Ceftriaxone	21.66±0.57^g^	12.33±0.57^c^	22.33±0.57^g^	15.33±0.57^e^	13.66±0.57^d^	16.66±0.57^f^	12.33±0.57^c^	8.33±0.57^a^	9.33±0.57^b^
Cefoxitin	14.33±0.57^e^	16.33±0.57^f^	13.66±0.57^e^	12.33±0.57^d^	10.33±0.57^c^	14.66±0.57^e^	8.66±0.57^b^	10.33±0.57^c^	7.33±0.57^a^
Levofloxacin	14.66±0.57^e^	10.33±0.57^c^	6.33±0.57^a^	17.33±0.57^f^	12.33±0.57^d^	7.33±0.57^a^	6.66±0.57^a^	8.66±0.57^b^	9.33±0.57^b^
Tetracycline	11.66±0.57^e^	9.33±0.57^c^	8.33±0.57^c^	11.33±0.57^e^	4.33±0.57^a^	8.33±0.57^c^	10.33±0.57^d^	8.66±0.57^c^	5.66±0.57^b^
Doxycycline	10.66±0.57^f^	6.66±0.57^c^	8.66±0.57^e^	10.33±0.57^f^	9.66±0.57^f^	6.33±0.57^c^	7.66±0.57^d^	5.33±0.57^b^	3.66±0.57^a^
Oxytetracycline	8.33±0.57^cde^	7.66±0.57^cd^	5.33±0.57^b^	7.33±0.57^c^	4.33±0.57^a^	11.66±0.57^f^	9.33±0.57^e^	8.66±0.57^de^	13.33±0.57^g^
Kanamycin	16.33±0.57^f^	14.33±0.57^e^	9.33±0.57^b^	10.66±0.57^c^	13.33±0.57^d^	8.66±0.57^b^	10.33±0.57^c^	14.66±0.57^e^	7.66±0.57^a^
Gentamicin	8.66±0.57^a^	10.66±0.57^b^	13.66±0.57^d^	7.66±0.57^a^	11.33±0.57^b^	12.66±0.57^c^	8.66±0.57^a^	10.66±0.57^b^	11.66±0.57^b^
Neomycin	13.33±0.57^e^	10.33±0.57^c^	14.33±0.57^f^	12.66±0.57^de^	9.66±0.57^c^	11.66±0.57^d^	8.33±0.57^b^	5.66±0.57^a^	12.33±0.57^de^
Streptomycin	13.33±0.57^f^	8.33±0.57^cd^	9.33±0.57^d^	10.33±0.57^e^	8.33±0.57^cd^	7.33±0.57^c^	5.33±0.57^b^	3.66±0.57^a^	7.66±0.57^c^
Bacitracin	10.33±0.57^d^	11.66±0.57^e^	8.33±0.57^c^	12.33±0.57^e^	9.33±0.57^cd^	3.66±0.57^a^	4.66±0.57^ab^	3.66±0.57^a^	5.33±1.00^b^
Chloramphenicol	18.33±0.57^g^	15.33±0.57^f^	13.33±0.57^d^	14.33±0.57^e^	10.66±0.57^c^	8.33±0.57^b^	6.66±0.57^a^	9.33±0.57^b^	8.33±0.57^b^
Norfloxacin	18.66±0.57^f^	14.33±0.57^d^	10.33±0.57^a^	12.66±0.57^c^	17.66±0.57^e^	13.33±0.57^c^	10.66±0.57^a^	17.33±0.57^e^	11.66±0.57^b^
Ciprofloxacin	17.33±0.57^a^	31.33±0.57^h^	24.33±0.57^d^	20.33±0.57^b^	22.66±0.57^c^	28.33±0.57^f^	22.33±0.57^c^	29.33±0.57^g^	26.33±0.57^e^

a–g Means within a row having different letters differ (p<0.05).
